# Can spontaneous pneumothorax be resolved in COVID-19 without hospital care? A case report

**DOI:** 10.22088/cjim.12.0.368

**Published:** 2021

**Authors:** Narges Tamaskani, Mahmoud Khandashpour, Somayeh Livani

**Affiliations:** 1 1.Clinical Research Development Center (CRDC), Sayad Shirazi Hospital, Golestan University of Medical Sciences, Gorgan, Iran

**Keywords:** COVID-19, Pneumothorax, Radiology, CT scan, Iran

## Abstract

**Background:**

We encountered the novel coronavirus infection as a pandemic in 2020. The infection started in Wuhan, China, and spread rapidly all over the world. CT scan has been used as an important diagnostic method in the detection of suspicious patients. One of the uncommon complications of coronavirus disease 2019 (COVID-19) is pneumothorax.

**Case presentation:**

A 47-year-old smoker male with COVID-19 diagnosis, good general condition and no respiratory complaint, complicated by pneumothorax. He refused hospitalization. After educating him about the red flags and quarantine protocols, he continued treatment at home .Cap amoxicillin/clavulanic acid 625mg was prescribed for one week. A follow-up CT represented only small involvement of lungs. Pneumothorax was resolved spontaneously without any medical intervention and hospitalization. O_2_ saturation was in normal range an there was no dry cough anymore.

**Conclusion:**

According to our clinical experience, pneumothorax is resolved spontaneously in a COVID-19 case. Considering general status and hemodynamic stability, it is suggested to reduce invasive interventions in COVID-19 cases with pneumothorax.

The role of imaging in controlling the epidemic of coronavirus disease-2019 (COVID-19) caused by SARS-CoV-2, has been dominant at the forefront of investigation for patients suspected to have COVID-19. Computed tomography (CT) of the chest has been performed on large scales to detect the typical features of the lung involvement in COVID-19 (1), including bilateral multilobar ground-glass opacification (GGO) with a peripheral or posterior distribution, apparent in the outer lateral zone of lungs (2, 3). Complications like pneumothorax has only been reported in about 1% of patients with COVID-19 (4). We, here reporting a middle-aged man with self-limiting pneumothorax treated as an outpatient.

## Case presentation

On March 13, 2020, a 47-year-old occasional-smoker man with unremarkable past medical history was admitted to our emergency department in the northeast Iran, with myalgia, dry cough, low-grade fever (37.9 °C), shivering and diaphoresis for the past 5 days. His vital signs were stable. He had no complaint of dyspnea and his O_2_saturation was 97%. There was no history of suspicious contact to a person with COVID-19. His blood sample results showed leukocytosis (19000 cells/μLiter; normal range 4000-10000 cells/μL) with 75 % neutrophil and 20% lymphocyte. C-reactive protein (CRP) was negative but erythrocyte sedimentation rate (ESR) was 25 mm/hour (normal range< 15 mm/hr). 

**Figure 1:**
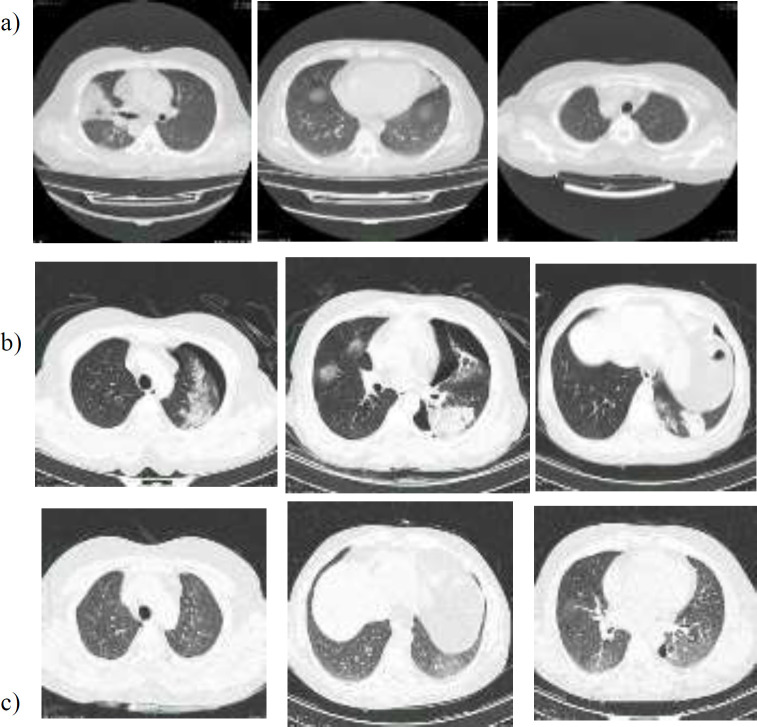
CT scan of a man with spontaneous pneumothorax due to the COVID-19

A non-contrast chest CT was done and showed a wedge-shaped consolidation with air bronchogram in the left upper lobe. Another small pleural based consolidation was also seen in the superior segment of the left lower lobe (figure 1.a). According to his good general condition and lack of dyspnea, he was discharged with outpatient orders and treated for COVID-19 infection by azithromycin for 5 days and chloroquine for 10 days. It should be considered that the diagnosis of COVID-19 was only based on the imaging due to the very shortage of PCR kits of COVID-19 at the beginning of the epidemic in Iran.

a) The first CT scan showed consolidation with air-bronchogram in the right upper lobe. b) The second CT showed left pneumothorax, patchy consolidation and patchy ground glass opacity with crazy paving. c) The third CT presented loculated pneumothorax and pleural thickening in left hemithorax and a subpleural band-like opacity in the posterior segment of basal lobes

During this period, he developed weakness and loss of appetite, with no sign of dyspnea or fever. On the 30^th^ of March, 2020 he fainted at home suddenly and experienced severe pain in left hemithorax. He was referred to the emergency department to rule out the cardiac angina. His electrocardiogram (ECG) and troponin level was normal. We performed the second CT at this time to distinguish any complication or progression of coronavirus infection that was encountered with left pneumothorax. Patchy consolidation with air bronchogram was seen in the left upper and lower lobe and right lower lobe. Patchy ground-glass opacity with superimposed septal thickening (crazy paving pattern) was seen in the right upper lobe too (figure 1.b). His O2 saturation was 98% and no sign of respiratory distress was detected and chronic obstructive pulmonary disease (COPD) was ruled out. The only symptom was a pain in the left hemithorax. The patient refused hospitalization; therefore, we sent the patient home notifying him of the red flags and quarantine protocols. Cap amoxicillin/clavulanic acid 625mg was prescribed for one week. 

One week later his general condition got better, and only complained from minimal pain in left hemithorax. We performed an x-ray and saw a little pneumothorax. He was in a good shape with normal O_2_ saturation, so he was sent home with azithromycin for one month. On May 11, 2020, a follow-up CT represented a small (20×11 mm) loculated pneumothorax and pleural thickening in the medial basal part of the left hemithorax. A subpleural band-like opacity was also seen in the anterior portion of left lower lobe and right upper lobe, and patchy ground-glass opacity was more predominant in the left side (figure 1.c). His general condition was good with O_2 _saturation of 98% and the dry cough has been resolved.

## Discussion

As the pandemic of COVID-19 progressed around the world, physicians encountered different complications and atypical presentations of the disease. One of the rare complications is pneumothorax that has been reported in the course of treatment with different consequences (5-6). The pathophysiology underlying the occurrence of the secondary spontaneous pneumothorax in COVID-19 proposed to increase alveolar pressure due to persistent coughing and alveolar rupture secondary to alveolar membrane damaged by the virus (7, 8). 

Our patient’s imaging showed same findings with previous reports of COVID-19 cases with spontaneous pneumothorax. as we observed in our case, consolidation and ground-glass opacities were also reported in previous clinical studies (9). In our patient, pneumothorax has been resolved without hospital admission or surgical intervention. Although in other cases, tube thoracostomy is needed to drain excess air (10). The need for chest tube was reported in all 6 cases of COVID-19 with spontaneous pneumothorax and among them 4 patients were associated with mechanical ventilation in another study from the USA (9). This scenario likely represents a rare and maybe a benign finding in coronavirus infection and it would be better not to heist for chest-tube insertion in the management of this complication, however, a close follow-up and patient’s collaboration are necessary in this regard. 

Although the explanation of the mechanism for this phenomenon is blurred but its self-limitation in our case could be accounted as a hint for further investigations about how spontaneous pneumothorax can be resolved in COVID-19 without hospital care.

 In conclusion our clinical experience showed pneumothorax can be resolved spontaneously in a COVID-19 case without hospitalization. Considering this case, it is then suggested in such cases with stable status to reduce invasive interventions. 
